# Prevalence and associated factors of sexually transmitted infections among methamphetamine users in Eastern China: a cross-sectional study

**DOI:** 10.1186/s12879-021-06987-8

**Published:** 2022-01-04

**Authors:** Xing Ye, Fu-Rong Li, Qing Pan, Zhen Li, Gong-Qi Yu, Hong Liu, Jian Liu, Peng-Cheng Huai, Fu-Ren Zhang

**Affiliations:** Shandong Provincial Hospital for Skin Diseases & Shandong Provincial Institute of Dermatology and Venereology, Shandong First Medical University & Shandong Academy of Medical Sciences, 27397 Jingshi Road, Jinan, 250022 Shandong China

**Keywords:** Methamphetamine, STIs, Prevalence, Associated factors

## Abstract

**Background:**

The reported incidence of sexually transmitted infections (STIs) in China has been increasing over the last decades, especially among drug users, which has become one of the main burdens of public health in China. This study was conducted to estimate the prevalence and associated factors of STIs among non-injecting methamphetamine (MA) users in Eastern China.

**Methods:**

A cross-sectional survey was conducted among 632 MA users in Eastern China in 2017. Demographic characteristics, sexual behaviors, behaviors of MA use and sexual health knowledge were collected through questionnaire. First pass urine specimens were collected and detected for deoxyribonucleic acid (DNA) of Neisseria gonorrhoeae (NG) and Chlamydia trachomatis (CT) with Nucleic Acid Amplification Technology (NAAT), while blood specimens were collected and detected for antibodies of Human immunodeficiency virus (HIV), Herpes simplex virus type-2 (HSV-2), and syphilis with enzyme-linked immune sorbent assay (ELISA).

**Results:**

Among the 632 MA users, 464 (73.42%) were males, 60.92% were < 35 years of age, 546 (86.39%) were Shandong residents. 317 (50.16%, 95% CI 46.26–54.06%) participants were tested positive for at least one kind of STIs, including 242 (38.29%, 95% CI 34.50–42.08%) for HSV-2, 107 (16.93%, 95% CI 14.01–19.85%) for active syphilis, 46 (7.28%, 95% CI 5.25–9.31%) for treated syphilis, 40 (6.33%, 95% CI 4.43–8.23%) for CT, 6 (0.95%, 95% CI 0.19–1.71%) for HIV, and 3 (0.47%, 95% CI 0.06–1.00%) for NG infection. 99 (15.66%, 95% CI 12.83–18.49%) participants were co-infected with two kinds of STIs, including 91 (14.40%, 95% CI 11.66–17.14%) participants were co-infected with HSV-2 and syphilis. 14 (2.22%, 95% CI 1.07–3.37%) participants were co-infected with three kinds of STIs, and 4 HIV positive participants were co-infected with both syphilis and HSV-2. In the multiple logistic regression analysis, the results showed that females (adjusted OR [AOR] = 7.30, 95% CI 4.34–12.30) and individuals ≥ 35 years of age (AOR = 2.97, 95% CI 2.04–4.32) were more likely to test positive for STIs among MA users, whereas participants who acquired sexual health knowledge primarily from the Internet (AOR = 0.57, 95% CI 0.40–0.82) and those whose regular partners did not use drugs (AOR = 0.59, 95% CI 0.37–0.94) were less likely.

**Conclusions:**

This study found that the prevalence of HSV-2 and syphilis are alarming high among non-injecting MA users in Shandong Province in Eastern China. The prevention and control intervention of STIs among MA users in Shandong were needed, especially on females and MA users ≥ 35 years of age.

## Background

According to the World Drug Report 2020, there are about 27 million amphetamines users (including amphetamine, methamphetamine, and pharmaceutical stimulants) worldwide [1]. In recent years, methamphetamine (MA) has replaced heroin as the most abused drug in China. The number of MA users in China has continued to expand at a breakneck pace in the past decade. The China National Narcotic Control Commission data showed that MA users accounted for 27.0% (360 thousand people) of registered drug users in China as at 2009 [2]. However, the number has dramatically increased to 1.19 million by the end of 2019, accounting for 55.21% of the 2.15 million registered drug users in the country [3]. MA is a highly addictive psychostimulant, which may intensify emotions, increase energy, and heighten sexuality. Compared with some other stimulant drugs such as cocaine, MA has a longer half-life and remains psychomotor active for longer [4, 5]. In addition, the synthetic processes for MA are refined on an ongoing basis to produce purer, more potent, and fashionable forms, at lower cost [1]. According to the relevant laws and regulations in China, the punishment for MA use is much lighter than for the use of traditional drugs, such as heroin. For example, the first time people are using MA they are only be fined and subjected to a few days of administrative detention, whereas those caught using heroin are immediately sentenced to at least 2 years of compulsory detoxification. Therefore, the use of MA may continue to increase in the years to come.

The burden of sexually transmitted infections (STIs) in China has been increasing within the last decades. The incidence of syphilis, Neisseria gonorrhoeae (NG), Human immunodeficiency virus (HIV), the three notifiable STIs, has increased from 33.09 cases per 100,000 residents in 2009 to 51.91 cases per 100,000 residents in 2019, with an average annual increase of 4.61% [6, 7]. And the incidence of the Chlamydia trachomatis (CT) and Herpes simplex virus type-2 (HSV-2) cases reported from 105 sexually transmitted surveillance sites between 2008 and 2017 has increased from 40.78 cases per 100,000 residents to 51.91 cases per 100,000 residents, expanding with an average annual increase of 2.71% [8–10]. However, these figures only reflect the tip of the iceberg because of the large missed diagnosis and underreporting in both notifiable-based and surveillance-based STIs reports [11].

According to findings of published studies, frequent use of methamphetamine has neurotoxic effects on the dopaminergic and serotonergic systems, leading to potentially irreversible loss of nerve terminals and/or neuron cell bodies [12–14]. In addition, some significant psychiatric withdrawal symptoms following abrupt cessation after periods of regular use, such as anhedonia, hypersomnia, irritability, anxiety, aggression, and intense cravings for methamphetamine have also been reported [14, 15]. Studies have also showed that the use of MA accelerates the spread of STIs [15]. MA use is closely related to various high-risk sexual behaviors, such as engaging in commercial sex work to obtain money for drugs, having a higher number of sexual partners, and having unprotected vaginal and/or anal sex [16]. In addition, the MA user population could form a “bridge group” after being STIs, and facilitate the transmission of STIs to non-drug using population [17–20]. Moreover, STIs are associated with an increased risk for HIV infection, causing an increased susceptibility to the human body to HIV virus [21–23].

The official data showed that there were 30 thousand registered drug users in Shandong province as at 2012 [24]. However, the number has strikingly increased to 104 thousand by the end of 2019 [25], expanding at an average annual growth rate of 19.44% (2012–2019). Shandong Province is an important economic center and maritime transportation hub on the Eastern Coast of China, attracting a large number of domestic and foreign investors and tourists. Due to a large population mobility, management challenges and potential risk caused by drug abuse remain rising. With the rapid development of economic and entertainment industry, the abuse and trafficking of MA in Shandong Province have become increasingly serious, especially in Qingdao, which is known as the “Iceland” [16].

Finding the epidemiological characteristics of STIs among MA users are essential for designing control program. To our knowledge, previous studies in China have been conducted with more focus on injecting drug users and other key population, only a few studies have focused on non-injecting MA users and estimated its impact on STIs in China [26–28]. However, similar studies conducted in other countries also have some limitations. For example, majority of the studies primarily have analyzed the related factors of STIs among injecting drug users (IDUs) or men who have sex with men (MSM), while the data for non-injecting MA users were scarce [29, 30]. It is therefore essential to conduct an updated study with a larger sample size that includes both males and females to estimate the prevalence of STIs among the MA users in China.

This study was conducted to estimate the prevalence and associated factors of STIs among non-injecting MA users in Eastern China to provide information for the formulation of targeted intervention to control the spread of STIs.

## Methods

### Participants and recruitment

A cross-sectional survey was conducted in three county-level cities of Shandong Province: Chengyang District in Qingdao city, Zhoucun District in Zibo city, and Qingzhou in Weifang city. Participants were recruited continuously from the Detention Center of Qingzhou Public Security Bureau, Shandong Women’s Detoxification Centre in Zhoucun, and Qingdao First Detention Center, from April, 11 to December, 5, 2017. A detention center (detention house) refers to a public security agency used for temporarily detaining a criminal suspect before the court makes a judicial decision for the suspected criminal suspect. According to the relevant laws and regulations in China, criminal suspects detained for drug offences and suspects arrested for other criminal offences are detained in detention centers. Inclusion criteria were as follows: (1) individuals ≥ 18 years of age; (2) using MA at least once a month in the past 6 months before being arrested, and having a positive result on the urine test; (3) being compulsorily detoxified by a public security agency owing to drug use; (4) voluntarily give informed consent to participate in the study, provide blood and urine specimens, and complete the questionnaire; (5) free of any psychiatric illness and learning disability during the survey. First, we calculated the sample size according to the following formula: a two-side inspection level α = 0.05 was required, and the expected prevalence of STIs among MA users was estimated to be 14% [31]. Within the tolerable margin of error of 2.8%, the planned randomized sample size was calculated as N = Z_α_^2^ * p * q/d^2^ = 1.96^2^ * 0.14*(1 − 0.14)/0.028^2^ = 590. Following this, cluster sampling was adopted to select the sample. We randomly selected two prison areas in each site, and all individuals in the selected area that met the inclusion criteria were included in our study. A total of 642 MA users were initially included in the study. However, 10 MA users were excluded because of ineligible blood (6 cases) and urine samples (4 cases), resulting in a final analytical sample of 632 participants.

### Data collection

Before this survey, fieldwork staff was trained by epidemiology and clinical laboratory professionals. The investigation team included two laboratory technicians for urine and blood collection, one male and one female questionnaire interviewers, and one financial staff for distribution of the interview allowance. In addition, one trained supervisor was designated for informed consent and quality control of the investigation process. The study was approved by the Ethics Committee of Shandong Provincial Institute of Dermatology and Venereology. Participants sat separately in a quiet room to ensure accuracy, privacy and anonymity of the information collected. Data collected from questionnaires that we designed included: demographic characteristics (gender, age, education, ethnicity, household registration, and how many MA users have you been acquainted with?), behaviors of MA use (reason for first MA use, frequency of MA use per week, primary site for MA use, whether have ever been infected with HIV?) and related sexual behaviors (primary sexual partner during MA use, whether had sex with multiple sexual partners during MA use? condom use in sex with multiple sexual partners during MA use, whether had sex with female sex workers (FSWs) during MA use? condom use in sex with commercial sexual partners during MA use, whether had sex with MSM during MA use? condom use in sex with MSM during MA use), and perception of sexual health knowledge (whether had a clear understanding of the routes of transmission of STIs? whether believed that it is possible for babies born to women with STIs to have STIs? whether believed that STIs would increase the risk of HIV transmission? whether acquired sexual health knowledge primarily from the Internet?). Demographic information was collected by face-to-face interviews, and the other information in the questionnaire was completed independently by the participants themselves to ensure confidentiality. “Have been acquainted with” was defined as they have met each other before and have contacts sometimes, but do not regularly meet. “During MA use” in “primary sexual partner during MA use” was defined as sexual encounters during a period of time when the participant was using MA.

### Laboratory detection

First pass urine specimens were collected from all participants with disposable urine preservation tube and detected for deoxyribonucleic acid (DNA) of NG and CT using Nucleic Acid Amplification Technology (NAAT) on cobas4800 platforms (Roche Molecular Systems, Inc.). Venous whole blood specimens were collected with 2 mL of vacuum EDTA anticoagulant blood tubes, and serums were recovered after centrifugation for detecting for HIV antibodies, HSV-2 human IgG class antibodies, and syphilis-specific antibodies with enzyme-linked immune sorbent assay (ELISA) procedure. To ensure the accuracy of the test result, ELISA-positive syphilis specimens underwent confirmatory testing with treponema pallidum particle assay (TPPA) and toluidine red unheated serum test (TRUST). Active syphilis was defined as both TPPA and TRUST positive, and treated syphilis as TPPA positive but TRUST negative. ELISA-positive HIV and HSV-2 specimens were repeat tested using the ELISA procedure for confirmation. According to the manufacture instructions, the sensitivity and specificity of the syphilis ELISA test were 96.70% and 100.00% respectively, whereas for the NAAT of CT and NG they were 96.6% and 100% respectively. The participants with positive results were notified in time by the local skin disease prevention and treatment health station or the centers for disease control and prevention (CDC). Patients were arranged to be treated by the prison doctor or recommended to the local hospital or CDC to receive standardized treatment under the guard of prison guards.

### Statistical analysis

Epidata 3.1 (EpiData association, Odense, Denmark) was used for data entry and validation and SAS 9.4 (SAS Institute Inc., Cary, NC, USA) for data management and analyses. Prevalence and 95% confidence interval (CI) of STIs were estimated based on the binomial distribution. Chi-square test was used to compare categorical variables between STIs positive and negative participants. Univariate analysis was performed to explore the association between STIs infection and related variables with logistic analysis and crude odds ratio (COR). All the variables with a *p*-value < 0.1 in univariate analysis that were biologically plausible and without missing values were included in the multivariate regression model. Multivariate analysis was performed by forward stepwise multiple logistic regression to control for confounding factors such as gender and age. In addition, multicollinearity among associated factors was examined using variance inflation factors, condition index, and variance proportions. Both adjusted odds ratio (AOR) and 95% CI were calculated for each explanatory variable in the final models. All statistical tests were two-sided, and variables with *p*-value < 0.05 were considered statistical significance.

## Results

### Demographic characteristics

We enrolled 632 MA users made up of 464 (73.42%) males, and 168 (26.58%) females, with a median age of 33.70 ± 8.62 years (range 18–69). 374 (59.18%) participants were single; 400 (63.29%) had only received middle school or a lower level of education; 93.67% were of Han descent; 232 (36.71%) were self-employed; 546 (86.39%) were Shandong residents; and nearly half of them (45.57%) have been acquainted with at least 10 MA users (Table 1).

**Table 1:** Demographic, behavioral characteristics, and perception of sexual health knowledge among MA users in Shandong, China

Variable	Proportion infected by STIs		χ^2^	*p-*value	COR (95% CI)
	n/N	n/N%			
1. Demographic characteristics					
Gender					
Male	181/464	39.01	86.80	< 0.01	Ref
Female	136/168	80.95			6.65 (4.33–10.19)
Age (years)					
< 35	171/385	44.42	12.99	< 0.01	Ref
≥ 35	146/247	59.11			1.81 (1.31–2.50)
Education					
Below primary school	38/54	70.37	8.63*	< 0.01	Ref
Primary school	41/80	51.25			0.44 (0.21–0.92)
Middle school	134/266	50.38			0.43 (0.23–0.80)
High/technical school	83/185	44.86			0.34 (0.18–0.66)
College or above	21/47	44.68			0.34 (0.15–0.77)
Marital status					
Married	136/258	52.71	1.14	0.29	Ref
Single/divorced/widowed	181/374	48.40			0.84 (0.61–1.16)
Ethnicity					
Han	294/592	49.66	0.92	0.34	Ref
Others	23/40	57.50			1.37 (0.72–2.62)
Employment					
Self-employed	113/232	48.71	0.31	0.58	Ref
Others	204/400	51.00			1.10 (0.79–1.52)
Household registration					
Shandong Province	261/546	47.80	8.91	< 0.01	Ref
Non-Shandong Province	56/86	65.12			2.04 (1.27–3.28)
How many MA users have you been acquainted with?					
< 10 persons	161/344	46.80	3.40	0.07	Ref
≥ 10 persons	156/288	54.17			1.34 (0.98–1.84)
2. Sexual behaviors					
Primary sexual partner during MA use					
Regular partner	199/365	54.52	7.92	0.02	Ref
Commercial sexual partner	30/59	50.85			0.86 (0.50–1.50)
Casual partner	88/208	42.31			0.61 (0.43–0.86)
Whether had sex with multiple sexual partners during MA use?					
No	207/418	49.52	0.20	0.65	Ref
Yes	110/214	51.40			1.08 (0.78–1.50)
Condom use in sex with multiple sexual partners during MA use^a^					
Always	16/43	37.21	5.31	0.07	Ref
Usually	67/127	52.76			1.88 (0.93–3.83)
Never	27/44	61.36			2.68 (1.13–6.37)
Whether changed condom when exchanging sex partners?^b^					
Always	46/98	46.94	–	0.67	Ref
Usually	32/64	50.00			1.13 (0.60–2.12)
Never	5/8	62.50			1.88 (0.43–8.32)
Whether had sex with FSWs during MA use?^c^					
No	78/216	36.11	1.43	0.23	Ref
Yes	103/248	41.53			1.26 (0.86–1.83)
Whether exchanged sex for drugs or money?^d^					
No	84/103	81.55	0.06	0.80	Ref
Yes	52/65	80.00			0.91 (0.41–1.99)
Condom use in sex with commercial sexual partner during MA use^e^					
Always	73/158	46.20	1.72	0.19	Ref
Never or usually	80/149	53.69			1.35 (0.86–2.12)
Whether had sex with MSM during MA use?^c^					
No	165/427	38.64	0.30	0.58	Ref
Yes	16/37	43.24			1.21 (0.61–2.39)
Condom use in sex with MSM during MA use^f^					
Always	5/8	62.50	–	0.25	Ref
Never or usually	11/29	37.93			0.37 (0.07–1.85)
Impact of duration of sexual episodes during MA use					
No change	83/128	64.84	17.08	< 0.01	Ref
Decrease	18/52	34.62			0.29 (0.15–0.57)
Increase	216/452	47.79			0.50 (0.33–0.75)
Whether regular sex partners know of your behavior of MA use?					
Yes	180/320	56.25	9.62	< 0.01	Ref
No	137/312	43.91			0.61 (0.45–0.83)
Whether regular sex partners use drugs?					
Yes	129/177	72.88	50.78	< 0.01	Ref
No	188/455	41.32			0.26 (0.18–0.38)
3. Behaviors of MA use					
Reason for first MA use					
Curiosity	191/400	47.75	11.17	0.05	Ref
Excitement and fashion	43/96	44.79			0.88 (0.57–1.39)
Deceived	28/51	54.90			1.33 (0.74–2.39)
Influenced by relatives and friends	16/26	61.54			1.75 (0.78–3.95)
Heavy stress of life	23/38	60.53			1.68 (0.85–3.31)
Others	16/21	76.19			3.50 (1.26–9.74)
Frequency of MA use per week					
≥ 1 time	218/427	51.05	0.42	0.52	Ref
< 1 time	99/205	48.29			0.90 (0.64–1.25)
Primary site for MA use					
At own or friends’ home	242/456	53.07	5.55	0.02	Ref
Hotels, dance halls, etc	75/176	42.61			0.66 (0.46–0.93)
Whether have ever been infected with HIV?					
Yes	7/8	87.50	4.52	0.03	Ref
No	310/624	49.68			0.14 (0.02–1.15)
4. Perception of sexual health knowledge					
Whether had a clear understanding of the routes of transmission of STIs?					
Yes	154/270	57.04	8.92	< 0.01	Ref
No	163/362	45.03			0.62 (0.45–0.85)
Whether it is possible for babies born to women with STIs to have STIs?					
Yes	237/424	55.90	16.97	< 0.01	Ref
No	80/208	38.46			0.49 (0.35–0.69)
Whether believed that STIs increase the risk of HIV transmission?					
Yes	211/401	52.62	2.66	0.10	Ref
No	106/231	45.89			0.76 (0.55–1.06)
Whether acquired sexual health knowledge primarily from television or broadcasts?					
No	151/294	51.36	0.32	0.57	Ref
Yes	166/338	49.11			0.91 (0.67–1.25)
Whether acquired sexual health knowledge primarily from newspapers or books?					
No	151/295	51.19	0.23	0.63	Ref
Yes	166/337	49.26			0.93 (0.68–1.27)
Whether acquired sexual health knowledge primarily from medical personnel?					
No	210/439	47.84	3.10	0.08	Ref
Yes	107/193	55.44			1.36 (0.97–1.91)
Whether acquired sexual health knowledge primarily from health publicity materials?					
No	200/375	53.33	3.72	0.05	Ref
Yes	117/257	45.53			0.73 (0.53–1.01)
Whether acquired sexual health knowledge primarily from family?					
No	281/578	48.62	6.44	0.01	Ref
Yes	36/54	66.67			2.11 (1.17–3.81)
Whether acquired sexual health knowledge primarily from friends?					
No	232/464	50.00	0.02	0.89	Ref
Yes	85/168	50.60			1.02 (0.72–1.46)
Whether acquired sexual health knowledge primarily from school education?					
No	259/515	50.29	0.02	0.89	Ref
Yes	58/117	49.57			0.97 (0.65–1.45)
Whether acquired sexual health knowledge primarily from the Internet?					
No	208/369	56.37	13.68	< 0.01	Ref
Yes	109/263	41.44			0.55 (0.40–0.76)

*MA* methamphetamine, *STIs* sexually transmitted infections, *HIV* human immunodeficiency virus, *COR* crude odds ratio, FSWs female sex workers, MSM men who have sex with men

*Mantel–Haenszel χ^2^

^a^Only for those who have had sex with multiple sexual partners during MA use, n = 214

^b^Only for those who use condoms when they had sex with multiple sexual partners during MA use, n = 170

^c^Only males, n = 464

^d^Only females, n = 168

^e^Only for those who have had sex with commercial sexual partner during MA use, n = 307

^f^Only for those who have had sex with MSM during MA use, n = 37

### MA use, sexual behaviors, and perception of sexual health knowledge

Of the participants, 67.56% (n = 427) reportedly used MA more than once per week. Majority (72.15%) of participants chose their own or friends’ home as their regular MA use site. In addition, 8 (1.27%) participants admitted being infected with HIV.

Of the 632 participants, nearly one-third (32.91%) of participants reported that their primary sexual partners during MA use were casual partners. About 33.86% (n = 214) of participants reported they have had sex with multiple sexual partners during MA use, but only 20.09% (n = 43) reported consistent condoms use. More than half (53.45%) of male participants have had sex with FSWs during MA use, whereas about 38.69% (n = 65) of the females reported they had exchanged sex for drugs or money. However, among the 307 participants who had engaged in commercial sex, only 51.47% (n = 158) reported consistent condom use. 7.97% (n = 37) the male participants admitted that they have had sex with MSM during MA use, but only 21.62% (n = 8) of them were reported consistent condom use. Majority (71.52%) of the participants thought that the duration of sexual episodes during MA use was increased. Nearly half (49.37%) of the participants reported that their regular partners did not know of their behavior of MA use, and regular partners of majority (71.99%) of the participants did not use drugs.

Several questions examined perception of sexual health knowledge among participants. Less than half (42.72%) of participants had a clear understanding of the routes of transmission of STIs, nearly one-third (32.91%) of participants reported that it is possible for babies born to women with STIs to have STIs, and 231 (36.55%) participants believed that STIs would not increase the risk of HIV transmission. Furthermore, among all the participants, 338 (53.48%) acquired sexual health knowledge primarily from television or broadcasts, 337 (53.32%) primarily from newspapers or books, 193 (30.54%) primarily from medical personnel, 257 (40.66%) primarily from health publicity materials, 54 (8.54%) from family, 168 (26.58%) primarily from friends, 117 (18.51%) primarily from school education, and 263 (41.61%) primarily from the Internet.

### Prevalence and associated factors of STIs among MA users

Among the 632 participants, 317 (50.16%, 95% CI 46.26–54.06%) were positive for at least one kind of STIs, including 242 (38.29%, 95% CI 34.50–42.08%) for HSV-2, 107 (16.93%, 95% CI 14.01–19.85%) for active syphilis, 46 (7.28%, 95% CI 5.25–9.31%) for treated syphilis, 40 (6.33%, 95% CI 4.43–8.23%) for CT, 6 (0.95%, 95% CI 0.19–1.71%) for HIV, and 3 (0.47%, 95% CI 0.06–1.00%) for NG infection. 99 (15.66%, 95% CI 12.83–18.49%) participants were co-infected with two kinds of STIs, including 91 (14.40%, 95% CI 11.66–17.14%) participants were co-infected with HSV-2 and syphilis. 14 (2.22%, 95% CI 1.07–3.37%) participants were co-infected with three kinds of STIs, and 4 HIV positive participants were co-infected with both syphilis and HSV-2 (Table 2). With the exclusion of 37 MSM from the male participants and 65 FSWs from the female participants, the prevalence of HSV-2 among non-MSM males and non-FSW females was 27.40% (95% CI 23.17–31.63%) and 70.87% (95% CI 62.10–79.64%), respectively.

**Table 2:** Prevalence of STIs among MA users in Shandong, China

	Male	Female	n	Prevalence (%)^a^	95% CI
STIs	181	136	317	50.16	46.26–54.06
HSV-2	124	118	242	38.29	34.50–42.08
Syphilis	86	67	153	24.21	20.87–27.55
Active (TPPA+ and TRUST+)	55	52	107	16.93	14.01–19.85
Treated (TPPA+ and TRUST−)	31	15	46	7.28	5.25–9.31
CT	23	17	40	6.33	4.43–8.23
HIV	0	6	6	0.95	0.19–1.71
NG	1	2	3	0.47	0.06–1.00
Co-infection	52	61	113	17.88	14.89–20.87
2 kinds of STIs	51	48	99	15.66	12.83–18.49
HSV-2 and syphilis	48	43	91	14.40	11.66–17.14
HSV-2 and CT	3	3	6	0.95	0.19–1.71
HSV-2 and NG	0	2	2	0.32	0.12–0.76
3 kinds of STIs	1	13	14	2.22	1.07–3.37
HSV-2 and syphilis and CT	1	9	10	1.58	0.61–2.55
HSV-2 and syphilis and HIV	0	4	4	0.63	0.01–1.25

^a^Estimated the total prevalence of each STIs

Results of the univariate analysis are shown in Table 1. The STIs prevalence was significantly higher in females (80.95%) than males (39.01%) (χ^2^ = 86.80, *p* < 0.01). Significant differences in prevalence of STIs were observed in terms of both age (χ^2^ = 12.99, *p* < 0.01) and educational level (χ^2^ = 11.51, *p* = 0.02). Positive for STIs was less among MA users < 35 years of age (44.42%) than ≥ 35 years of age (59.11%), and highest among participants with below primary school education (70.37%). In addition, prevalence of STIs also significantly differed by household registration (χ^2^ = 8.91, *p* < 0.01), and was less among Shandong residents (47.80%) than non-Shandong (65.12%).

The prevalence of STIs was highest among those who have had sex primarily with their regular partner during MA use (54.52%) (χ^2^ = 7.92, *p* = 0.02) and those who thought that the duration of sexual episodes during MA use has no change (64.84%) (χ^2^ = 17.08, *p* < 0.01). Significant differences in STIs prevalence were observed between whether their regular partners knew of their behavior of MA use (χ^2^ = 9.62, *p* < 0.01), and whether their regular partners used drugs (χ^2^ = 50.78, *p* < 0.01). The prevalence among participants who chose their own or friends’ home (53.07%) as their regular MA use site was higher than those who chose hotels, guesthouse, dance halls, nightclubs, etc. (42.61%) (χ^2^ = 5.55, *p* = 0.02), who admitted they had been infected with HIV was higher than those denied (χ^2^ = 4.52, *p* = 0.03), who had a clear understanding of the routes of transmission of STIs (55.90%) was higher than those did not know (38.46%) (χ^2^ = 8.92, *p* < 0.01), and higher among those thought that it is possible for babies born to women with STIs to have STIs (55.90%) than those had an opposite view (38.46%) (χ^2^ = 16.97, *p* < 0.01). Moreover, STIs prevalence also differed statistically by primary ways of acquiring sexual health knowledge. Participants that acquired sexual health knowledge primarily from the Internet had the lowest prevalence (41.44%) (χ^2^ = 13.68, *p* < 0.01), while primarily from health propaganda (45.53%) (χ^2^ = 3.72, *p* = 0.05) and family (66.67%) (χ^2^ = 6.44, *p* = 0.01) had higher prevalence.

Table 3 shows the results of the multivariate logistic regression analysis for the related factors found statistically significant in the univariate analysis. The results revealed that females (AOR = 7.30, 95% CI 4.34–12.30) and individuals ≥ 35 years of age (AOR = 2.97, 95% CI 2.04–4.32) were more likely to test positive for STIs among MA users, whereas participants who acquired sexual health knowledge primarily from the Internet (AOR = 0.57, 95% CI 0.40–0.82) and those whose regular partners did not use drugs (AOR = 0.59, 95% CI 0.37–0.94) were less likely.

**Table 3:** Multivariate analysis on risk factors related to STIs among MA users in Shandong, China

Variable	STIs positive (N = 317)		STIs negative (N = 315)		COR (95% CI)	AOR (95% CI)	*p-*value
	n	n/N%	n	n/N%			
Gender							< 0.01
Male	181	57.10	283	89.84	Ref	Ref	
Female	136	42.90	32	10.16	6.65 (4.33–10.19)	7.30 (4.34–12.30)	
Age (years)							< 0.01
< 35	171	53.94	214	67.94	Ref	Ref	
≥ 35	146	46.06	101	32.06	1.81 (1.31–2.50)	2.97 (2.04–4.32)	
Whether acquired sexual health knowledge primarily from the Internet?							< 0.01
No	208	65.62	161	51.11	Ref	Ref	
Yes	109	34.38	154	48.89	0.55 (0.40–0.76)	0.57 (0.40–0.82)	
Whether regular sex partners use drugs?							0.03
Yes	129	40.69	48	15.24	Ref	Ref	
No	188	59.31	267	84.76	0.26 (0.18–0.38)	0.59 (0.37–0.94)	

*STIs* sexually transmitted infections, *COR* crude odds ratio, *AOR* adjust odds ratio

## Discussion

The STIs epidemic in China is characterized by major concentration of cases in key population groups including MSM, FSWs, drug users, migrant workers, and youth [32]. The present study was conducted to investigate the prevalence of STIs and its associated factors among MA users in Eastern China. We found a STIs prevalence of 50.16% (95% CI 46.26–54.06%) among MA users, and the three main STIs with highest prevalence in this population were HSV-2 (38.29%, 95% CI 34.50–42.08%), followed by syphilis (24.21%, 95% CI 20.87–27.55%), and CT (6.33%, 95% CI 4.43–8.23%). The prevalence of HSV-2 in our study was equivalent to the prevalence (33.9%, 95% CI 27.2–41.2%) reported by Oster AM among injecting drug users aged 18–59 from NHANES (1999–2010) in the United States [33]. And the prevalence of HSV-2 among female MA users (80.95%, 95% CI 75.01–86.89%) in our study is not significantly higher than the prevalence (72.06%, 95% CI 67.92–76.20%) reported by Duan in Shandong province in 2015 [34]. With the exclusion of 37 MSM from the male participants and 65 FSWs from the female participants, the prevalence of HSV-2 among non-MSM males and non-FSW females were 27.40% (95% CI 23.17–31.63%) and 70.87% (95% CI 62.10–79.64%), respectively. Comparatively, a previous cross-sectional survey in six Chinese cities showed that the prevalence of HSV-2 was about 12.07% (95% CI 10.97–13.17%) among MSM [35]. Another meta-analysis in China among FSWs found the prevalence of HSV-2 was about 15.80% (95% CI 11.70–20.90%) [36]. In both non-human animal and human studies, MA use has been reported to increase physical sexual desire and psychological pleasure, which is associated with high-risk sexual behaviors such as unprotected sex, and group sex [37–39]. Moreover, compared with MSM and FSWs who did not use MA, MA users are more likely to report high-risk sexual behaviors such as anal or vaginal sex, condomless sex and group sex, and it is reported that drug use will result in prolonged sexual activity, which does present an increased risk of transmission of STIs [40, 41]. Therefore, we have reason to believe that the prevalence of STIs among MA users may be higher than that among MSM and FSWs.

In the present study, the multiple logistic regression analysis revealed that gender of MA users was significantly associated with the prevalence of STIs. Female MA users were more likely to be infected by STIs, as the prevalence of STIs in females was 7.30 times higher (AOR = 7.30, 95% CI 4.34–12.30) than in males. This may be due to a variety of factors. First, in our sample population, when compared with male MA users, female MA users were more likely to report that their primary sexual partner during MA use was their regular partner (45.47% vs. 91.67%). Several studies reported that use of condoms with a regular partner was less frequent than with a casual partner, because condom use with regular partners was viewed more often as a sign of disrespect and a barrier to intimacy and affection, rather than as a positive means of showing respect [42–44]. Second, female MA users in the current study seem to have been less likely to report that they had had sex with multiple sexual partners during MA use, when compared with male MA users (25.00% vs. 37.07%). However, female MA users were more likely than males to admit inconsistent condom use with multiple sexual partners (83.33% vs. 79.07%). Moreover, about 38.69% (65/168) of female MA users have been involved in commercial sex for drugs or money, and 35.37% (23/65) of these females reported inconsistent use of condoms, causing 19 of them to have been infected with STIs. However, only 7.97% (37/464) of males reported having had sex with MSM, and 78.38% (29/37) of them had inconsistent condom use, causing 11 of them to be infected with STIs. In some studies, users reported that MA could heighten their libido and prolong their sexual climax, but impair their judgment, and therefore they had sex with more sexual partners, forgot condom use, and were unaware of condom breakage. It is biologically plausible that there is an increased risk of STIs resulting from tissue damage associated with prolonged unprotected intercourse [41, 45]. In addition, there is significant evidence suggesting that female sex hormones such as estrogen and progesterone could contribute to increased acquisition of STIs in women during exposure to viral STIs [46]. All these differences in behaviors and biology between male and female MA users remind us that efforts to reduce STIs should recognize the positive aspects of female MA users.

Our findings also found that MA users ≥ 35 years of age may be related to the risk of STIs. Increased odds of seropositivity of HSV-2 and syphilis with age would be expected, given that the risk of ever being infected by STIs would accumulate over time, especially for those with more sexual partners and having unprotected encounters. In addition, previous research found that older participants had an increased risk of HIV infection in Cambodia [47]. A possible explanation is that MA has significant effects on both innate and adaptive immune responses, increasing the damage to the immune system over the duration of MA use and rendering users susceptible to STIs [48, 49]. As most STI prevention-oriented materials and assessments are currently aimed at younger persons, we suggest that future STI prevention activities may need to be supplemented with interventions that specifically target person over 35 years of age.

The current study also found that the ways to acquire sexual health knowledge have an important impact on SITs among MA users. Logistic regression model showed that the acquisition of sexual health knowledge primarily from the Internet may be a protective factor. These suggested that compared with traditional media such as TV and broadcasts, the Internet may better cater for MA users [50]. The effect of health education on them from the Internet is more effective, and it is particularly important for them to master sexual health knowledge, so as to control the spread of STIs more effectively. Additionally, those whose regular sex partners did not use drugs tend to be less likely to test positive for STIs, maybe due to the risks of unprotected sexual behaviors between them were reduced, and the risks of STIs were also at reduced.

There were several limitations to this study. First, this study was conducted in Shandong, a province of Eastern Coast of China. Therefore, our findings should be interpreted with caution as they may not be applicable to MA users in other areas of China. Second, prevalence of STIs among individuals less than 18 years old and older than 69 years old was not estimated in our study. Hence, further study to investigate the prevalence of STIs among these population is needed. Third, the data collection of this study relied on self-reporting and involved some sensitive questions which may cause recall bias and information bias. In spite of these limitations, our data still present some basis for the development of targeted interventions.

## Conclusions

This study found that the prevalence of HSV-2 and syphilis are alarming high among non-injecting MA users in Shandong Province in Eastern China. The prevention and control intervention of STIs among MA users in Shandong were needed, especially for females and MA users ≥ 35 years of age.

## Data Availability

In order to protect the privacy of the participants, the collected data has not been made publicly available. The data are available from the corresponding author upon reasonable request.

## Abbreviations

MA: Methamphetamine
STIs: Sexually transmitted infections
NG: *Neisseria gonorrhoeae*
HIV: Human immunodeficiency virus
CT: *Chlamydia trachomatis*
HSV-2: Herpes simplex virus type-2
IDUs: Injecting drug users
MSM: Men who have sex with men
FSWs: Female sex workers
CI: Confidence interval
COR: Crude odds ratio
AOR: Adjusted odds ratio
